# Effect of Puerarin, Baicalin and Berberine Hydrochloride on the Regulation of IPEC-J2 Cells Infected with Enterotoxigenic* Escherichia coli*

**DOI:** 10.1155/2019/7438593

**Published:** 2019-02-12

**Authors:** Xiaoxi Liu, Fenghua Liu, Yunfei Ma, Huanrong Li, Xianghong Ju, Jianqin Xu

**Affiliations:** ^1^China Agricultural University-Beijing University of Agriculture Traditional Chinese Veterinary Medicine Teaching and Research Team, College of Veterinary Medicine, China Agricultural University, Beijing 100193, China; ^2^Department of Veterinary Medicine, Guangdong Ocean University, Zhanjiang 524088, China; ^3^Beijing Key Laboratory for Dairy Cow Nutrition, College of Animal Science and Technology, Beijing University of Agriculture, Beijing 102200, China

## Abstract

Puerarin, baicalin and berberine hydrochloride are the main components of Gegen Qinlian Decoction, which has been used to treat diarrhoea in China for hundreds of years, yet the biological function and molecular mechanism of these components are not clear. To investigate the effects of puerarin, baicalin, and berberine hydrochloride on the regulation of porcine intestinal epithelial cells (IPEC-J2 cells) infected with enterotoxigenic* Escherichia coli *(ETEC). IPEC-J2 cells were pretreated with puerarin (200 *μ*g/mL), baicalin (1 *μ*g/mL), and berberine hydrochloride (100 *μ*g/mL) at 37°C for 3 h and then coincubated with the F4ac ETEC bacterial strain 200 at 37°C for 3 h. ETEC infection damaged the structure of IPEC-J2 cells, upregulated* mucin 4* (*P* < 0.01) and* mucin 13* mRNA (*P* < 0.05) expression, increased the apoptosis rate (*P* < 0.05), and promoted inflammatory responses (*IL-6* and* CXCL-2 *mRNA expression) in IPEC-J2 cells by activating the nuclear factor-*κ*B (NF-*κ*B) signaling pathway. Pretreatment with puerarin, baicalin, and berberine hydrochloride improved the structure and morphology of IPEC-J2 cells and inhibited ETEC adhesion by downregulating specific adhesion molecules. Pretreatment with baicalin decreased the inflammatory response; pretreatment with baicalin and berberine hydrochloride decreased the inflammatory response mediated by the NF-*κ*B signaling pathway. Pretreatment with puerarin, baicalin, and berberine hydrochloride protected IPEC-J2 cells from ETEC infection by inhibiting bacterial adhesion and inflammatory responses.

## 1. Introduction

As an important etiological factor of postweaning diarrhoea, enterotoxigenic* Escherichia coli* (ETEC) induces severe diarrhea in postweaned piglets and causes great economic loss to the swine industry [1, 2]. The colonization factors which mediate bacterial adherence to intestinal epithelial cells are essential to the process of diarrhea [2, 3]. ETEC adheres to intestinal epithelial cells after binding to specific receptors [4], producing heat-stable enterotoxins (ST, including STa and STb) and heat-labile enterotoxin, which disrupt host cell functions, stimulate fluid and electrolyte secretion, and eventually cause diarrhea [2, 5, 6].

ETEC infection would induce the dysbiosis of gut microbiota in mice [7], trigger autophagy in IPEC-1 cells [8], and promote the expression of proinflammatory cytokines through NF-*κ*B and MAPK pathways in the jejunum of mice [9]. As a striking candidate gene of the F4ac (F4 fimbriae antigenic variant) ETEC receptor, mucin 4 (Muc4) contributes to the immune system as a major component of the glycocalyx layer of epithelial cells [10, 11]. Mucin 13 (Muc13) is also an important adhesion molecule in mucosal epithelial signaling in response to* E. coli* in pigs [12]. To counter ETEC invasion, the intestinal epithelium activates multiple innate defense mechanisms [13]; microarray clustered terms of differentially expressed genes in porcine intestinal epithelial cells (IPEC-J2) infected with F4ac ETEC were shown to be mainly involved in apoptosis and inflammatory responses [11]. Apoptosis is a form of programmed cellular death that can be activated through either extrinsic or intrinsic pathways [14]. ETEC infection and STb toxin have been shown to induce apoptosis in intestinal epithelial cells [14, 15]. The mucosal immune system detects pathogen-associated molecular patterns by membrane-bound Toll-like receptors (TLRs), and signaling via TLRs leads to the production of proinflammatory cytokines, chemokines, and antimicrobial peptides, which triggers innate immune and adaptive immune responses [3, 16].

Gegen Qinlian Decoction, as described in the Treatise on Febrile Diseases (Shang Han Lun), a classic resource of traditional Chinese medicine written by Zhongjing Zhang (150–215 AD), is commonly used to treat diarrhoea, enteritis, diabetes, coronary heart disease, and general fever in clinical practice for hundreds of years [17–19]. Gegen Qinlian Decoction can be used to treat the postweaning diarrhoea as the theory of traditional Chinese veterinary medicine, but the molecular mechanism of this decoction is not clear. Puerarin, baicalin, and berberine hydrochloride are its main components [17]. As an isoflavonoid, puerarin derives from* Puerariae Radix* [roots of* Pueraria lobata* (Willd.) Ohwi (Ge Gen)]; it exhibits a wide spectrum of pharmacological properties such as cardioprotection, neuroprotection, antioxidant and anti-inflammatory activities, and alleviation of pain [20]. Baicalin is a flavonoid extracted from the* Scutellariae Radix* [roots of* Scutellaria baicalensis *(Huang Qin)]; it has the potential to minimize inflammation and protect injured decidual cells and intestinal epithelial cells [21–23]. Berberine hydrochloride is the hydrochloride of berberine (a kind of natural isoquinoline alkaloid), which is isolated from Chinese herbs* Coptidis Rhizoma* [rhizomes of* Coptis chinensis *Franch. (Huang Lian)] [24]. Berberine has long been used to treat gastrointestinal infections and diarrhea and exerts its antidiarrheal effect partially by enhancing the absorption of Na^+^ and water [25, 26]. It has been reported Gegen Qinlian decoction was used to treat acute colitis efficiently [27]; however, the molecular mechanism of Gegen Qinlian Decoction in the treatment of ETEC-induced diarrhea in piglets is unclear, and little is known about the effects of puerarin, baicalin, and berberine hydrochloride on the regulation of intestinal epithelial cells. The purpose of this study was therefore to investigate the effects of puerarin, baicalin, and berberine hydrochloride on the regulation of IPEC-J2 cells infected with ETEC.

## 2. Methods

### 2.1. Cell and Bacterial Strain Culture

Porcine intestinal cells (IPEC-J2) were grown in 1:1 Dulbecco's modified eagle medium (DMEM)/Ham's F-12 medium (GIBCO, Grand Island, NY, USA) containing 10% fetal bovine serum (FBS, #10099-141, GIBCO, Grand Island, USA), 50 IU/mL penicillin, and 50 mg/mL streptomycin and incubated at 37°C under 5% (v/v) CO_2_. The medium was replaced 48 h after initial cell plating. F4ac ETEC strain 200 (O149:K91:F4ac, LT+, STa+, STb+, and EAST1+) bacteria were removed from cryostorage and cultured in Ordinary Broth Agar at 37°C for three generations (24 h per generation).

### 2.2. Cell Viability Assay

Puerarin, baicalin, and berberine hydrochloride can be found in the pharmacopeia of China. Puerarin (C_21_H_20_O_9_, purity: 95.5%), baicalin (C_21_H_18_O_11_, purity: 93.3%), and berberine hydrochloride (C_20_H_18_ClNO_4_, purity: 86.7%) were purchased from the National Institutes for Food and Drug Control (Beijing, China), where the content of these three active compounds had been determined as standard substances. To measure cell viability, equivalent numbers of IPEC-J2 cells were plated on 96-well plates (Corning Inc., New York, USA) and cultured in DMEM/F12 medium containing 10% FBS. After reaching 95% confluence, cells were washed twice with phosphate buffered saline (PBS), serum-starved for 2 h, and treated with various concentrations of puerarin (25–400 *μ*g/mL), baicalin (1 ng/mL–10 *μ*g/mL), and berberine hydrochloride (10–200 *μ*g/mL) for 12 h or 24 h. Next, 10 *μ*L of CCK-8 solution (water-soluble tetrazolium) (Dojindo, Kumamoto, Japan) was added to each well and the plate was incubated for 1 h at 37°C. Absorbance was measured at 450 nm using a microplate reader (Bio-Rad Laboratories Inc., Foster City, CA, USA).

### 2.3. Infection of IPEC-J2 Cells

For cell infection experiments, ETEC bacteria were subcultured in DMEM/F12 medium at 37°C for 3 h, centrifuged, and washed with sterile PBS (pH 7.4). Next, a bacterial suspension (2 × 10^6^ colony-forming units [CFU]/mL, multiplicity of infection [MOI] = 10:1) was prepared in PBS and bacteria were treated with puerarin (200 *μ*g/mL), baicalin (1 *μ*g/mL), and berberine hydrochloride (100 *μ*g/mL) for 3 h before bacterial total RNA was extracted. Control cells were maintained at 37°C under 5% CO_2_. In pretreatment groups, IPEC-J2 cells at 95% confluence were washed twice with PBS and then treated with puerarin (200 *μ*g/mL), baicalin (1 *μ*g/mL), and berberine hydrochloride (100 *μ*g/mL) at 37°C, 5 % CO_2_ for 3 h. A total of 20 mL of ETEC bacterial suspension (2 × 10^6^ CFU/mL, MOI = 10:1) was added to the ETEC infection group, puerarin pretreatment group, baicalin pretreatment group, and berberine hydrochloride pretreatment group (5 mL per group). IPEC-J2 cells and bacteria were coincubated at 37°C for 3 h and the various groups were used in subsequent experiments.

### 2.4. Examination of Scanning Electron Microscopy (SEM)

Cells were fixed with 2% electron microscopy-grade glutaraldehyde for 2 h, fixed in 1% osmium tetroxide with 0.1% potassium ferricyanide, dehydrated using an ethanol gradient (30%–90%), and embedded in EPON epoxy resin. Ultrathin sections (65 nm) were prepared, stained with 2% uranyl acetate coated with gold layer, and examined under a scanning electron microscope (Hitachi, Tokyo, Japan). After image formation, the number of single bacteria attached to the IPEC-J2 cells was collected with 3 independent pictures.

### 2.5. Examination of Transmission Electron Microscopy (TEM)

Cells were collected and centrifuged at 3000 g for 15 min, fixed with 4% glutaraldehyde for 12 hs, and post-fixed in cold 1% osmium tetroxide. Cells were then dehydrated in a graded acetone series and embedded in epoxy resin. Ultrathin sections were stained with saturated uranyl acetate in 50% ethanol and lead citrate and then examined with a JEM-1230 transmission electron microscope (JEOL, Tokyo, Japan). After image formation, the rate of swollen mitochondria/normal mitochondria and the number of lysosomes were accounted with 3 independent pictures.

### 2.6. RNA Extraction and Quantitative RT-PCR

Total RNA was isolated from IPEC-J2 cells using a phenol and guanidine isothiocyanate-based TRIzol reagent (Invitrogen, Carlsbad, CA, USA) as described in the manufacturer's instructions. Polymerase chain reaction (PCR) mixtures (20 *μ*L in total) were prepared to contain 10 *μ*L of SYBR Green PCR mix (Stratagene, La Jolla, CA, USA), 0.3 *μ*L of reference dye, 1 *μ*L of each primer (10 *μ*mol/L), 1 *μ*L of cDNA template, and 6.7 *μ*L of diethylpyro-carbonate-treated water. The cycling conditions were as follows: 95°C for 3 min followed by 40 cycles of 95°C for 10 s, 60°C for 20 s, and 72°C for 60s. Quantitative PCR analysis was carried out using the DNA Engine Mx3000P® (Agilent, Santa Clara, CA, USA) fluorescence detection system against a double-stranded DNA-specific fluorescent dye (Stratagene, La Jolla, CA, USA) according to optimized PCR protocols. Expression levels were determined using the relative threshold cycle (C_t_) method as described by the manufacturer. Reverse transcription-generated cDNA encoding* β-actin*,* MUC4*,* MUC13*,* IL-1α*,* IL-6*,* CXCL-2*, and* PLAU* was amplified by real-time PCR using selective primers (Table 1, which should appear at this location). For each cellular RNA sample, *β*-actin was amplified in parallel with the target genes and used as a normalization control.

### 2.7. Flow Cytometric Analysis

To measure apoptosis, cells were treated with ETEC according to the previously described experimental procedure. Following each specific treatment, cells were treated with 0.25% trypsin (GIBCO, Grand Island, NY, USA), washed twice with PBS, and stained with annexin V/propidium iodide (Nanjing Jiancheng Bioengineering Institute, Nanjing, China). Flow cytometric analysis was performed using cell count according to the manufacturer's instructions (BD Biosciences, San Jose, CA).

### 2.8. Western Blot Analysis

Proteins from IPEC-J2 cells were extracted using a total protein extraction kit (BioChain Institute Inc., Hayward, CA, USA) and a nuclear and cytoplasmic protein extraction kit (KeyGEN BioTECH, Nanjiang, Jiangsu, China). Proteins were quantified with a BCA protein assay kit (Pierce Biotechnology Inc., Rockford, IL, USA). Proteins (10 *μ*g/sample) were separated by SDS-PAGE (Invitrogen Inc.), transferred to nitrocellulose membranes (88585, Pierce, Rockford, USA), and then hybridized with the specific antibodies. The following antibodies used were as follows: I*κ*B*α* (#4814, Cell Signaling Technology, Danvers, MA, USA), NF-*κ*B p65 (#6956, Cell Signaling Technology, Danvers, MA, USA), Phospho-NF-*κ*B p65 (#3033, Cell Signaling Technology, Danvers, MA, USA), *β*-Actin (#4970, Cell Signaling Technology, Danvers, MA, USA), and Lamin A/C (#4777T, Cell Signaling Technology, Danvers, MA, USA). Total protein and cytoplasmic protein blots were normalized by using *β*-actin to correct for differences in loading of the proteins. Nuclear protein blots were normalized by using lamin A/C to correct for differences in loading of the proteins. Proteins were detected using an Odyssey Infrared Imaging System (LI-COR Biosciences, Lincoln, NE, USA). Densitometric values of immunoblot signals were obtained from three separate experiments using ImageJ software (National Institutes of Health, Bethesda, MD, USA).

### 2.9. Immunofluorescence and Confocal Microscopy

IPEC-J2 cells were washed three times with PBS and then blocked with 5% bovine serum in PBS containing 0.2% Triton X-100 for 1 h. The cells were then incubated with the primary antibody Phospho-NF-*κ*B p65 (P-P65) (Rabbit IgG, #3033, Cell Signaling Technology, Danvers, MA, USA) overnight at 4°C. After washing three times with PBS, biotin-conjugated donkey anti-rabbit IgG (#711067003, Jackson) and streptavidin Alexa Fluor 594-conjugated antibody (#S11227, Invitrogen) were consecutively added and incubated for 1 h at room temperature. Finally, nucleus morphology was visualized using DAPI (#236276, Roche) with washing cells for three times. Images of IPEC-J2 cells were performed on a confocal microscope (Nikon, Tokyo, Japan). Representative image stacks of average intensity from relevant channels were merged and converted into red and blue format. All related groups were subjected to the same color settings for each color channel. Fluorescence intensities of P-P65 in the nucleus of IPEC-J2 cells were determined using the ImageJ software (National Institutes of Health, Bethesda, MD, USA).

### 2.10. Statistical Analysis

Data were analyzed using Student's t-test when appropriate or by a one-way analysis of variance (ANOVA) and Tukey's multiple comparison tests were applied when comparing more than three means; the results were presented as the mean ± SEM, with values of* P* < 0.05 considered statistically significant. Statistical analyses were carried out using the SPSS12.0 software (Inc., and IBM Company, Chicago, USA) and graphs were created using Origin 6.0 (National Institutes of Health, NY, USA).

## 3. Results

### 3.1. Cytotoxicity of Puerarin, Baicalin, and Berberine Hydrochloride in IPEC-J2 Cells

**Figure 1:**
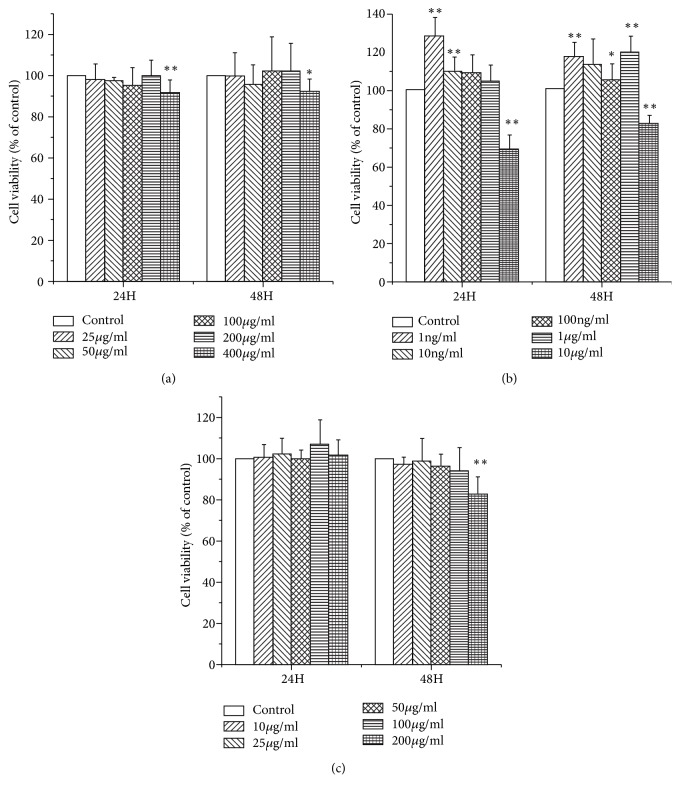
*Cytotoxicity of puerarin, baicalin, and berberine hydrochloride in IPEC-J2 cells*. Treatment with puerarin at 200 *μ*g/mL for 48 h did not significantly change the viability of IPEC-J2 cells (a). Treatment with baicalin at concentrations from 1 ng/mL to 1 *μ*g/mL markedly promoted IPEC-J2 cell proliferation without causing cytotoxicity (b). At concentrations of 100 *μ*g/mL or less berberine hydrochloride was found to be safe for treating IPEC-J2 cells for 48 h (c). Data are mean ± SEM, n = 6 per treatment (*∗P* < 0.05 versus control group; *∗∗P* < 0.01 versus control group).

To select appropriate concentrations of puerarin, baicalin, and berberine hydrochloride for treating IPEC-J2 cells, cells were exposed to various concentrations of these agents for 24 h or 48 h before cell viability was determined. Treatment with puerarin at 200 *μ*g/mL for 48 h did not significantly change the viability of IPEC-J2 cells (Figure 1(a)). After being stimulated with 400 *μ*g/mL of puerarin for 24h (*P* < 0.01) and 48h (*P* < 0.05), cell viabilities were significantly inhabited (Figure 1(a)). It indicated puerarin had no cytotoxic effect on IPEC-J2 cells under the concentration of 200 *μ*g/mL. Treatment with baicalin concentrations ranging from 1 ng/mL to 1 *μ*g/mL markedly promoted IPEC-J2 cell proliferation without causing cytotoxicity (Figure 1(b)). After being stimulated with 10 *μ*g/mL of baicalin for 24h (*P* < 0.01) and 48h (*P* < 0.01), cell viabilities were significantly decreased (Figure 1(b)). At concentrations of 100 *μ*g/mL or less, berberine hydrochloride had no cytotoxic effect on IPEC-J2 cells for 48 h (Figure 1(c)). After being stimulated with 200 *μ*g/mL of berberine hydrochloride for 48h, cell viabilities were significantly reduced (*P* < 0.01) (Figure 1(c)). To investigate effects of puerarin, baicalin, and berberine hydrochloride on the regulation of IPEC-J2 cells, the maximum safety concentrations were selected for further research. Thus, puerarin at a concentration of 200 *μ*g/mL, baicalin at a concentration of 1 *μ*g/mL, and berberine hydrochloride at a concentration of 100 *μ*g/mL were selected in this study.

### 3.2. Morphological Ultrastructural Changes in IPEC-J2 Cells

**Figure 2:**
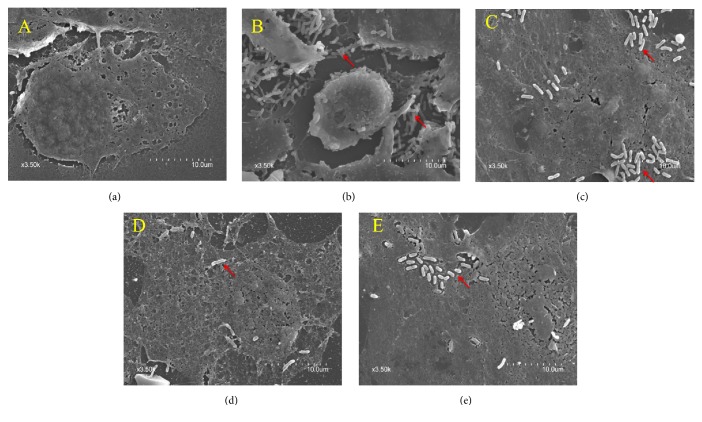
*Ultrastructural morphology of IPEC-J2 cells observed using scanning electron microscopy*. Under normal conditions, the structure of IPEC-J2 cells was intact (a). Following ETEC infection, a large number of ETEC cells adhered to the surface of IPEC-J2 cells. ETEC damaged the structure of IPEC-J2 cells and caused shrinking of cellular morphology (b). Pretreatment with puerarin at 200 *μ*g/mL (c), baicalin at 1 *μ*g/mL (d), and berberine hydrochloride at 100 *μ*g/mL (e) improved the structure and morphology of IPEC-J2 cells and decreased the number of adhered single bacteria ((a) control cells; (b) treatment with ETEC for 3 h; (c) pretreatment with puerarin 200 *μ*g/mL + ETEC; (d) pretreatment with baicalin 1 *μ*g/mL + ETEC; (e) pretreatment with berberine hydrochloride 100 *μ*g/mL + ETEC; red arrows represent the single bacteria of ETEC).

Using SEM, a large number of ETEC bacteria were shown to adhere to the surface of IPEC-J2 cells after ETEC infection (Figures 2(a)-2(b)). ETEC damaged the structure of IPEC-J2 cells and caused shrinking of cellular morphology (Figure 2(b)), while pretreatment with puerarin, baicalin, and berberine appeared to protect the structure and morphology of IPEC-J2 cells (Figures 2(c)–2(e)). Relative to the ETEC infection group alone, pretreatment with puerarin at 200 *μ*g/mL significantly decreased the number of ETEC attached to the IPEC-J2 cells 0.33-fold (Figure 4(a)), pretreatment with baicalin at 1 *μ*g/mL significantly decreased the number of attached ETEC 0.12-fold (Figure 4(a)), and pretreatment with berberine pretreatment at 100 *μ*g/mL significantly decreased the number of attached ETEC 0.21-fold (Figure 4(a)).

**Figure 3:**
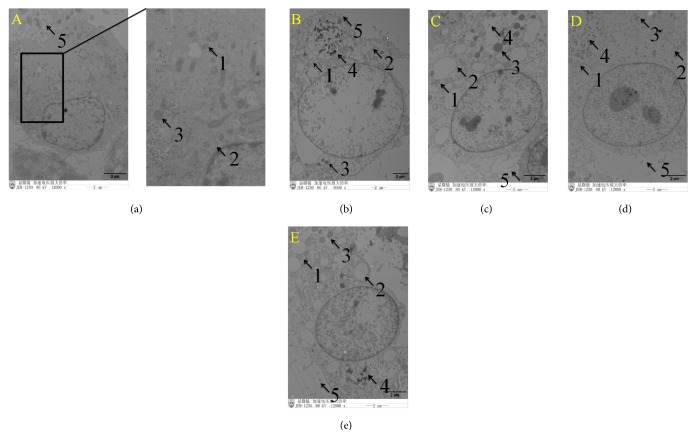
*Ultrastructural morphology of IPEC-J2 cells observed using transmission electron microscopy*. Under normal conditions, the structure of IPEC-J2 cells was intact (a). Following ETEC infection, epithelial cell microvilli were shed from the cellular surface. Mitochondria increased in size and became more spherical, mitochondrial matrixes became shallower, and mitochondrial vacuolization was observed. Endoplasmic reticulum increased in size (b). In the puerarin (c), baicalin (d), and berberine pretreatment (e) groups, swollen mitochondria were also observed, the endoplasmic reticulum increased in size, the number of lysosomes increased, and digested fragments of bacteria were observed in the cytoplasm. Pretreatment with baicalin (d) significantly improved the structure of IPEC-J2 cells. 1 represents mitochondria, 2 represents endoplasmic reticulum, 3 represents lysosome, 4 represents bacterial fragments, and 5 represents epithelial cell microvilli ((a) control cells; (b) treatment with ETEC for 3 h; (c) pretreatment with puerarin 200 *μ*g/mL + ETEC; (d) pretreatment with baicalin 1 *μ*g/mL + ETEC; (e) pretreatment with berberine hydrochloride 100 *μ*g/mL + ETEC).

**Figure 4:**
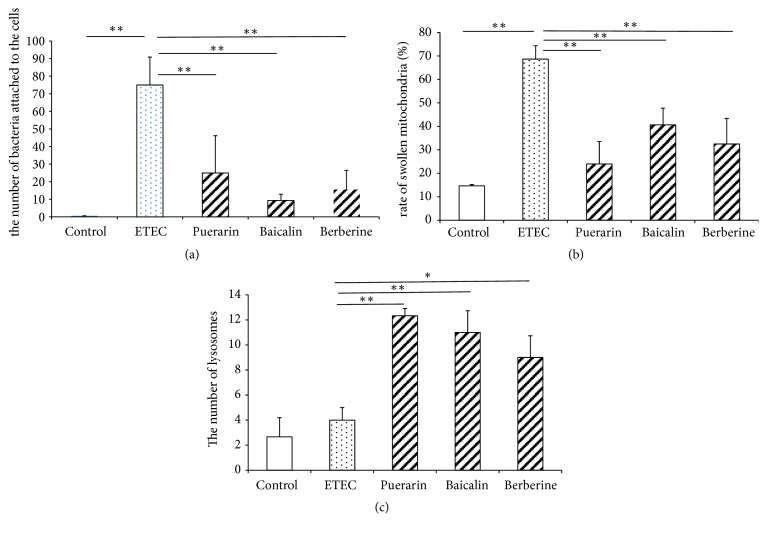
*Changes of the number of attached ETEC and Organelles in IPEC-J2 cells*. Effect of puerarin (200 *μ*g/mL), baicalin (1 *μ*g/mL), and berberine hydrochloride (100 *μ*g/mL) on the number of attached ETEC (a), the rate of swollen mitochondria / normal mitochondria (b) and the number of lysosomes (c) in IPEC-J2 cells infected with ETEC. Control: untreated cells; ETEC: treatment with ETEC for 3 h; puerarin: pretreatment with puerarin 200 *μ*g/mL + ETEC; baicalin: pretreatment with baicalin 1 *μ*g/mL + ETEC; berberine: pretreatment with berberine hydrochloride 100 *μ*g/mL + ETEC. Data are mean ± SEM, n = 3 per treatment (*∗P* < 0.05; *∗∗P* < 0.01).

Using TEM, ETEC infection caused shedding of epithelial cell microvilli. Furthermore, mitochondria increased in size and became more spherical, mitochondrial matrixes became shallower, mitochondrial vacuolization was observed, and the endoplasmic reticulum increased in size (Figure 3(b)). Pretreatment with baicalin significantly improved IPEC-J2 cell structure. In the puerarin, baicalin, and berberine pretreatment groups, mitochondrial swelling was also observed, the endoplasmic reticulum increased in size, and digested fragments of bacteria were observed in the cytoplasm (Figures 3(c)–3(e)). Relative to the control group, ETEC infection significantly increased the rate of swollen mitochondria/normal mitochondria 4.67-fold (Figure 4(b)). Relative to the ETEC infection group alone, pretreatment with puerarin at 200 *μ*g/mL significantly decreased the rate of swollen mitochondria/normal mitochondria 0.35-fold (Figure 4(b)), pretreatment with baicalin at 1 *μ*g/mL significantly decreased the rate 0.59-fold, and pretreatment with berberine pretreatment at 100 *μ*g/mL significantly decreased 0.47-fold (Figure 4(b)). ETEC infection did not significantly change the number of lysosomes. Relative to the ETEC infection group alone, pretreatment with puerarin at 200 *μ*g/mL significantly increased the number of lysosomes 3.1-fold (Figure 4(c)), pretreatment with baicalin at 1 *μ*g/mL significantly increased the number of lysosomes 2.75-fold (Figure 4(c)), and pretreatment with berberine pretreatment at 100 *μ*g/mL significantly increased the number of lysosomes 2.25-fold (Figure 4(c)).

### 3.3. mRNA Expression of Adhesion Molecules in IPEC-J2 Cells

**Figure 5:**
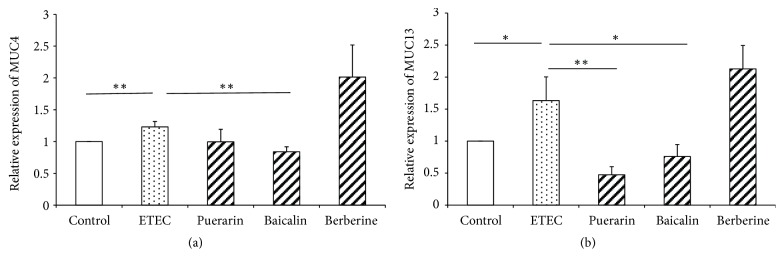
*mRNA expression of adhesion molecules in IPEC-J2 cells*. Effect of puerarin (200 *μ*g/mL), baicalin (1 *μ*g/mL), and berberine hydrochloride (100 *μ*g/mL) on* MUC4 *(a) and* MUC13 *(b) mRNA expression in IPEC-J2 cells infected with ETEC. Control: untreated cells; ETEC: treatment with ETEC for 3 h; puerarin: pretreatment with puerarin 200 *μ*g/mL + ETEC; baicalin: pretreatment with baicalin 1 *μ*g/mL + ETEC; berberine: pretreatment with berberine hydrochloride 100 *μ*g/mL + ETEC. Data are mean ± SEM, n = 3 per treatment (*∗P* < 0.05; *∗∗P* < 0.01).

ETEC infection significantly increased* MUC4* (*P* < 0.01) and* MUC13* (*P* < 0.05) mRNA expression in IPEC-J2 cells 1.23-fold and 1.63-fold (Figures 5(a)-5(b)), respectively. Relative to the ETEC infection group alone, pretreatment with puerarin significantly decreased the expression of* MUC13* 0.29-fold (*P* < 0.01) (Figure 5(b)). Pretreatment with baicalin at 1 *μ*g/mL significantly decreased the expression of* MUC4* (*P* < 0.05) and* MUC13* (*P* < 0.01) 0.68-fold and 0.47-fold (Figures 5(a)-5(b)), respectively.

### 3.4. Rate of Apoptosis in IPEC-J2 Cells

**Figure 6:**
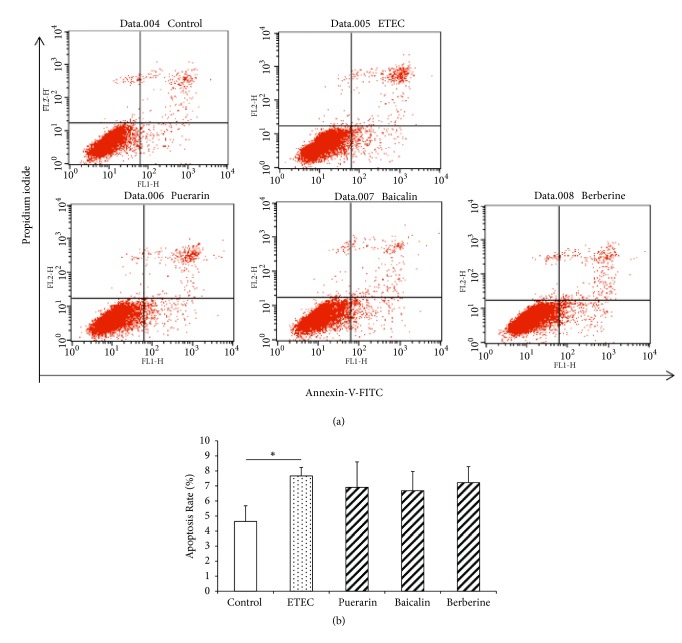
*Rate of apoptosis in IPEC-J2 cells*. (a) Apoptosis was detected in IPEC-J2 cells by flow cytometry. The normal rate of apoptosis in IPEC-J2 cells was 4.64%. ETEC infection for 3 h significantly increased apoptosis to 7.67%. (b) Pretreatment with baicalin and berberine hydrochloride did not significantly decrease the apoptosis. Control: untreated cells; ETEC: treatment with ETEC for 3 h; puerarin: pretreatment with puerarin 200 *μ*g/mL + ETEC; baicalin: pretreatment with baicalin 1 *μ*g/mL + ETEC; berberine: pretreatment with berberine hydrochloride 100 *μ*g/mL + ETEC. Data are mean ± SEM, n = 3 per treatment (*∗P* < 0.05; *∗∗P* < 0.01).

To determine the effect of puerarin, baicalin, and berberine hydrochloride on the regulation of cell death, the rate of apoptosis was measured using flow cytometry (Figure 6(a)). In this study, rate of apoptosis in IPEC-J2 cells is 4.64% (Figure 6(b)). ETEC infection for 3 h significantly increased the apoptosis rate of IPEC-J2 cells to 7.67% (Figure 6(b)). However, pretreatment with puerarin, baicalin, and berberine hydrochloride had no significant effect on the rate of apoptosis of IPEC-J2 cells (Figure 6(b)).

### 3.5. The Protein Level of I*κ*B*α*, P65, and P-P65

**Figure 7:**
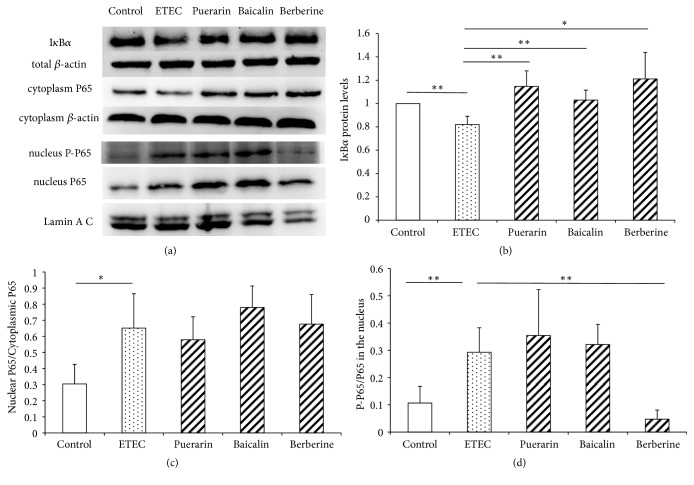
*The expression of proteins related to NF-κB signaling pathway in IPEC-J2 cells*. (a) Relative expression of I*κ*B*α*, total *β*-actin, cytoplasm P65, cytoplasm *β*-actin, nucleus P-P65, nucleus P65, and Lamin A/C in IPEC-J2 cells was assayed through western blot. (b–d) Quantitation of relative protein levels of I*κ*B*α*, cytoplasm P65/nucleus P65, and nucleus P-P65/ P65 in (a), respectively. Control: control cells without any processing; ETEC: treatment with ETEC for 3h; puerarin: pretreatment with puerarin at 200 *μ*g/mL + ETEC; baicalin: pretreatment with baicalin at 1 *μ*g/mL + ETEC; berberine hydrochloride: pretreatment with berberine hydrochloride at 100 *μ*g/mL + ETEC. Data are mean ± SEM, n = 3 per treatment (*∗P* < 0.05; *∗∗P* < 0.01).

Nuclear factor-*κ*B (NF-*κ*B) signaling pathway is important to the inflammatory responses. ETEC infection significantly decreased I*κ*B*α* protein expression in IPEC-J2 cells 0.80-fold (*P* < 0.01) (Figures 7(a) and 7(b)), significantly increased the ratio of nuclear P65/cytoplasmic P65 2.10-fold (*P* < 0.05) (Figures 7(a) and 7(c)), and significantly increased the nuclear P-P65/P65 protein level 2.73-fold (*P* < 0.01) (Figures 7(a) and 7(d)). It indicated ETEC infection for 3h had activated the NF-*κ*B pathway in IPEC-J2 cells. Relative to the ETEC infection group alone, pretreatment with puerarin, baicalin, and berberine hydrochloride did not block the translocation of P65 from cytoplasmic to nucleus. Relative to the ETEC infection group alone, pretreatment with baicalin at 1*μ*g/mL significantly increased I*κ*B*α* protein expression to 1.25-fold (*P* < 0.01) (Figures 7(a) and 7(b)) and significantly increased the nuclear P65 protein level 1.65-fold (*P* < 0.05). Relative to the ETEC infection group alone, pretreatment with berberine hydrochloride at 100*μ*g/mL significantly increased I*κ*B*α* protein expression 1.54-fold (*P* < 0.05) (Figures 7(a) and 7(b)) and significantly decreased the nuclear P-P65/P65 protein level 0.16-fold (*P* < 0.01) (Figures 7(a)–7(d)).

### 3.6. Localizaion of NF-*κ*B P65 and Phosphorylated Subunit P65 (P-P65) Proteins in the Nucleus

**Figure 8:**
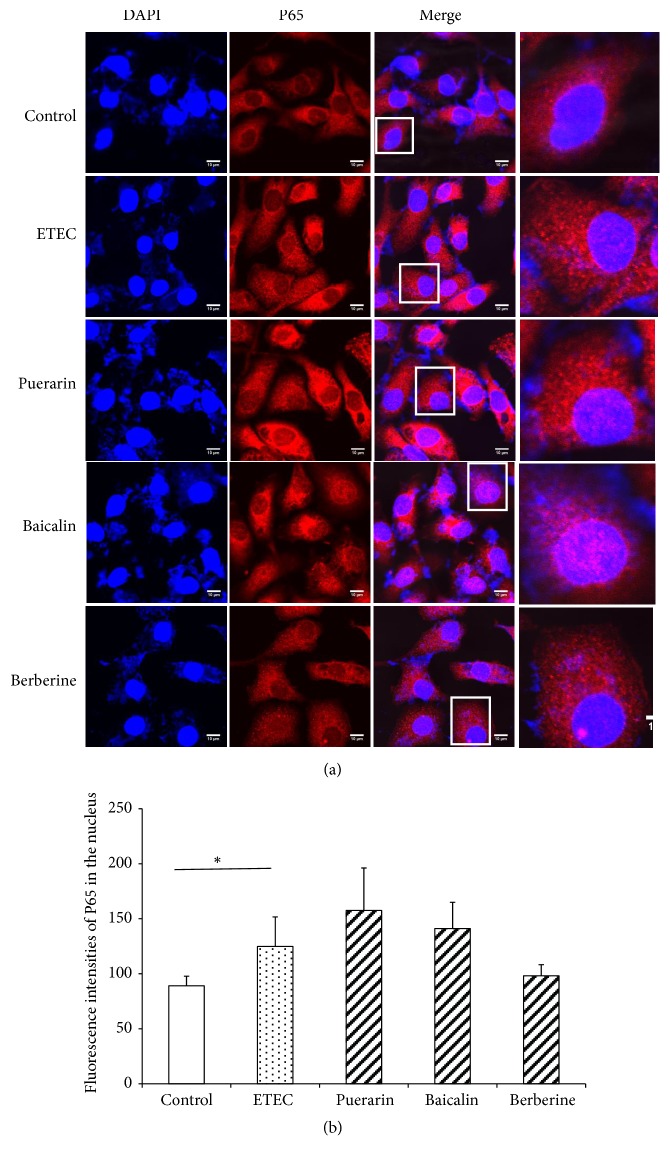
*Confocal microscopy of IPEC-J2 cells and quantification of fluorescence intensities of NF-κB P65 in the nucleus*. (a) Confocal microscopy of IPEC-J2 cells. The cells were stained for the nucleus (blue) and the protein NF-*κ*B P65 (red). Top row: untreated IPEC-J2 cells; second row: treatment with ETEC for 3 h; third row: pretreatment with puerarin 200 *μ*g/mL + ETEC; fourth row: pretreatment with baicalin 1 *μ*g/mL + ETEC; bottom row: pretreatment with berberine hydrochloride 100 *μ*g/mL + ETEC. Bar scale represents 10 *μ*m. (b) Quantification of fluorescence intensities of NF-*κ*B P65 in the nucleus. ETEC infection increased the fluorescence intensities of NF-*κ*B P65 in the nucleus. Relative to the ETEC infection group alone, pretreatment with baicalin and berberine hydrochloride significantly decreased the fluorescence intensities of NF-*κ*B P65 in the nucleus. Control: untreated cells; ETEC: treatment with ETEC for 3 h; puerarin: pretreatment with puerarin at 200 *μ*g/mL + ETEC; baicalin: pretreatment with baicalin at 1 *μ*g/mL + ETEC; berberine: pretreatment with berberine hydrochloride at 100 *μ*g/mL + ETEC. Data are mean ± SEM, n = 6 per treatment (*∗P* < 0.05).

**Figure 9:**
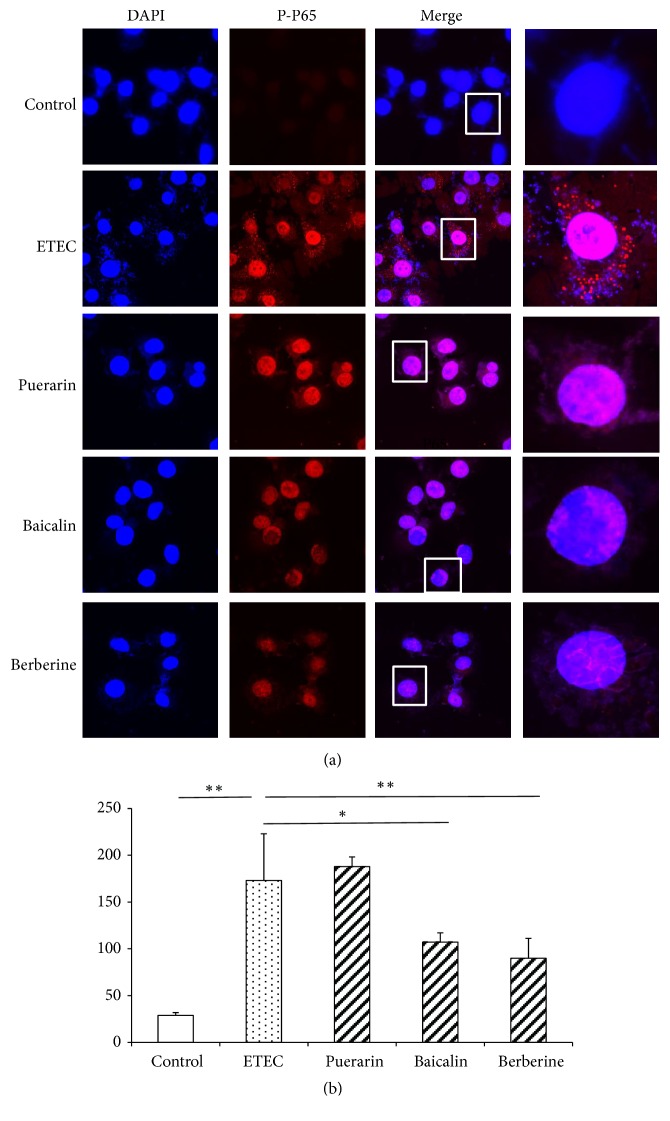
*Confocal microscopy of IPEC-J2 cells and quantification of fluorescence intensities of P-P65 in the nucleus*. (a) Confocal microscopy of IPEC-J2 cells. The cells were stained for the nucleus (blue) and the protein P-P65 (red). Top row: untreated IPEC-J2 cells; second row: treatment with ETEC for 3 h; third row: pretreatment with puerarin 200 *μ*g/mL + ETEC; fourth row: pretreatment with baicalin 1 *μ*g/mL + ETEC; bottom row: pretreatment with berberine hydrochloride 100 *μ*g/mL + ETEC. Bar scale represents 10 *μ*m. (b) Quantification of fluorescence intensities of P-P65 in the nucleus. ETEC infection increased P-P65 protein expression in the nucleus. Relative to the ETEC infection group alone, pretreatment with baicalin and berberine hydrochloride significantly decreased P-P65 protein expression in the nucleus. Control: untreated cells; ETEC: treatment with ETEC for 3 h; puerarin: pretreatment with puerarin at 200 *μ*g/mL + ETEC; baicalin: pretreatment with baicalin at 1 *μ*g/mL + ETEC; berberine: pretreatment with berberine hydrochloride at 100 *μ*g/mL + ETEC. Data are mean ± SEM, n = 6 per treatment (*∗P* < 0.05; *∗∗P* < 0.01).

Confocal microscopy showed NF-*κ*B P65 was expressed in the cytoplasm and nucleus of IPEC-J2 cells in normal condition (Figure 8(a)). ETEC infection significantly increased the fluorescence intensities of NF-*κ*B P65 in the nucleus 1.40-fold (*P* < 0.05) (Figure 8(b)), but pretreatment with puerarin, baicalin, and berberine hydrochloride did not significantly change the fluorescence intensities of NF-*κ*B P65 in the nucleus (Figure 8(b)). Confocal microscopy of IPEC-J2 cells showed that the fluorescence intensities of P-P65 in IPEC-J2 cells were weak in the normal condition (Figure 9(a)). After ETEC infection, P-P65 was strongly expressed in the cytoplasm and nucleus, and the fluorescence intensities of P-P65 in the nucleus were significantly increased 6.0-fold (*P* < 0.01) (Figure 9(b)). Pretreatment with puerarin, baicalin, and berberine hydrochloride focused most of P-P65 to the nucleus (Figure 9(a)), pretreatment with baicalin at 1 *μ*g/mL significantly decreased the fluorescence intensities of P-P65 in the nucleus of IPEC-J2 cells 0.62-fold (*P* < 0.05) (Figure 9(b)), and pretreatment with berberine hydrochloride at 100 *μ*g/mL significantly decreased the fluorescence intensities of P-P65 in the nucleus 0.52-fold (*P* < 0.01) (Figure 9(b)).

### 3.7. mRNA Expression of Inflammatory Cytokines in IPEC-J2 Cells

**Figure 10:**
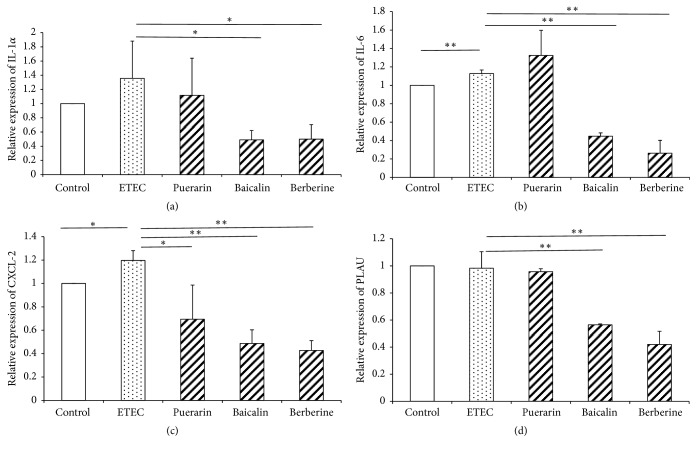
*mRNA expression of inflammatory cytokines in IPEC-J2 cells*. Effect of puerarin (200 *μ*g/mL), baicalin (1 *μ*g/mL), and berberine hydrochloride (100 *μ*g/mL) on* IL-1α *(a),* IL-6* (b),* CXCL-2 *(c), and* PLAU* (d) mRNA expression in IPEC-J2 cells infected with ETEC. Control: untreated cells; ETEC: treatment with ETEC for 3 h; puerarin: pretreatment with puerarin at 200 *μ*g/mL + ETEC; baicalin: pretreatment with baicalin at 1 *μ*g/mL + ETEC; berberine: pretreatment with berberine hydrochloride at 100 *μ*g/mL + ETEC. Data are mean ± SEM, n = 3 per treatment (*∗P* < 0.05; *∗∗P* < 0.01).

ETEC infection significantly increased* IL-6* (*P* < 0.01) and* CXCL-2* (*P* < 0.05) mRNA expression 1.13-fold and 1.20-fold (Figures 10(b)-10(c)), respectively, indicating that ETEC activated an inflammatory response in IPEC-J2 cells. Relative to the ETEC infection group alone, pretreatment with puerarin at 200 *μ*g/mL significantly decreased* CXCL-2* (*P* < 0.01) expression 0.58-fold (Figure 10(c)). Pretreatment with baicalin at 1 *μ*g/mL significantly decreased the expression of* IL-1α* (*P* < 0.05),* IL-6* (*P* < 0.01),* CXCL-2* (*P* < 0.01), and* PLAU* (*P* < 0.01) 0.36-fold, 0.40-fold, 0.41-fold, and 0.57-fold (Figures 10(a)–10(d)), respectively. Pretreatment with berberine hydrochloride at 100 *μ*g/mL significantly decreased the expression of* IL-1α* (*P* < 0.05),* IL-6* (*P* < 0.01),* CXCL-2* (*P* < 0.01), and* PLAU* (*P* < 0.01) 0.37-fold, 0.23-fold, 0.36-fold, and 0.43-fold (Figures 10(a)–10(d)), respectively. ETEC infection induced the cellular inflammation response of IPEC-J2, while pretreatment with baicalin and berberine hydrochloride reduced the inflammation response.

## 4. Discussion

There are more than 50 compounds in Gegen Qinlian Decoction [28]; however the content of most of these ingredients is very low. Due to their low concentrations, and the difficulty in studying the biological functions of all these 50 compounds at the same time, major active compounds were chosen as representatives for the further research. The pharmacokinetics of Gegen Qinlian Decoction after oral administration to rats showed the concentration of puerarin is the highest in the bioactive isoflavonoids of Puerariae Radix, as well as baicalin is* Scutellariae Radix* and berberine in* Coptidis Rhizoma* [19]. Because of their high concentrations and potential efficacy in Gegen Qinlian Decoction, puerarin, baicalin, and berberine hydrochloride were selected to coculture with IPEC-J2 cells in vitro.

In the present study, pretreatment with puerarin, baicalin, and berberine hydrochloride improved the cellular morphology and decreased the inflammatory response of IPEC-J2 cells. ETEC adherence is the first step of the whole process of invasion. Muc4 mediates immunity [10] while Muc13 has a protective role in the colonic epithelium and governs susceptibility towards ETEC F4ac in pigs [29, 30]. In this study, a large number of single bacteria adhered to the surface of IPEC-J2 cells, the expression of* MUC4* and* MUC13* mRNA was significantly increased after ETEC infection, and it is inferred that mutual promotion may exist between ETEC adherence and mucin expression. Compared with the ETEC infection group, pretreatment with puerarin significantly decreased the expression of* MUC13* mRNA, pretreatment with baicalin significantly decreased the expression of* MUC4 *and* MUC13 *mRNA, and few single bacteria were shown to adhere to the surface of IPEC-J2 cells in these two groups. Thus, pretreatment with puerarin and baicalin can interrupt the attachment of* E. coli* F4ac to IPEC-J2 cells by blocking mucin expression. Compared with the ETEC infection group, pretreatment with berberine hydrochloride decreased the number of single bacteria on the surface of IPEC-J2 cells, consistent with previous studies showing that berberine sulfate inhibited bacterial adherence to the mucosal or epithelial surface [31]. Maybe this effect can be attributed to the decreased expression of* ITGB5* mRNA (data not shown), which promotes the attachment of ETEC to porcine jejunal cells and maintains the epithelial barrier and immunity function [12].

In this study, ETEC infection caused damage to the structure of IPEC-J2 cells and enhanced the rate of apoptosis; however, the increased apoptosis rate in this condition might not have any physiological impact to cells. After ETEC infection, the microvilli of epithelial cells were shed. The anatomical structures of intestinal microvilli were extremely sensitive to hypoxia, and inflammatory conditions aggravated the low-pressure hypoxic state to increase damage to the microvilli [32]. It is referred that ETEC-induced inflammatory conditions cause damage to the structure of microvilli. ETEC strains induce water and electrolyte loss from the intestine. ETEC infection caused swelling of the mitochondria and endoplasmic reticulum, potentially because of the disrupted secretion of fluid and electrolytes. A previous study has shown that STb toxin induces a mitochondrial-mediated apoptotic pathway that acts as the main intrinsic pathway in rat ileum epithelial cells. Therefore, the damage to mitochondria and the enhanced rate of apoptosis can be attributed to STb secreted by ETEC [14]. In this study, pretreatment with puerarin, baicalin, and berberine hydrochloride for 3 h did not protect IPEC-J2 cells from apoptosis after ETEC infection, but mitochondrial swelling was observed in these groups. Mitochondrial swelling can also be observed when cuprous oxide nanoparticles induce the mitochondrial-mediated apoptosis of cancer cells, so it might be the characteristic of apoptosis. In this study, pretreatment with puerarin, baicalin, and berberine hydrochloride increased the number of lysosomes, which receive and degrade macromolecules from endocytic and phagocytic membrane-trafficking pathways [33]. Fragments of bacteria in the cytoplasm were also observed in these groups, showing that these compounds promoted the lysosome function of degrading ETEC bacteria in IPEC-J2 cells.

Previous studies showed that F4^+^ ETEC activated TLR4-mediated inflammatory responses upon recognition of lipopolysaccharide (LPS) by epithelial cells [3, 34]. The transcription factor NF-*κ*B, which induces the expression of numerous proinflammatory mediators such as cytokines and chemokines, is the final effector molecule of the TLR4 signaling pathway [32, 35]. Normally, NF-*κ*B dimers are retained in the cytoplasm by members of the inhibitor of *κ*B (I*κ*B) protein family and I*κ*B*α* is the principal inhibitory protein [32, 36]. After being activated, NF-*κ*B P65 is able to translocate to the nucleus where the phosphorylated subunit p65 plays an important role in triggering the transcription of NF-*κ*B target genes. IL-1 and IL-6, central elements of the cell-mediated immune response, are important inflammatory factors located downstream from NF-*κ*B [35, 37]. As one of the key regulators of leukocyte migration, CXCL2 encodes secreted proteins involved in immunoregulatory and inflammatory processes [38]. The anti-inflammatory mediator PLAU encodes a serine protease involved in the degradation of the extracellular matrix [39]. In this study, ETEC infection significantly decreases I*κ*B*α* protein expression, enhanced the P65 translocation from cytoplasm to nucleus, increased the protein level of P-P65/P65 in the nucleus, increased P65 and P-P65 fluorescence intensities in the nucleus, and increased the mRNA expression of* IL-6 *and* CXCL-2*, indicating ETEC infection activated the NF-*κ*B pathway and its downstream cellular inflammation response. It has been reported that ETEC SEC470 infection affected NF-*κ*B signaling and enhanced proinflammatory cytokine expression in the jejunum of mouse [9], which were in accordance with our study. However, different kinds of ETEC strains would cause opposed inflammatory responses. For example, ETEC H10407 infection inhibited the activation of NF-*κ*B in HCT-8 cells [40], and ETEC 298 inhibited proinflammatory cytokines expression in the jejunum of mice [41]. Compared with the ETEC infection group, pretreatment with baicalin decreased the mRNA expression of inflammatory cytokines (*IL-1α, IL-6, *and* CXCL-2*), whereas no significant changes were obtained for the translocation of P65 and the nuclear content of P-P65/P65, indicating pretreatment IPEC-J2 cells with baicalin at 1 *μ*g/mL cannot regulate the inflammatory responses of IPEC-J2 cells by the phosphorylation of P65 in the nucleus. However, baicalin (5×10^−5^ ~5×10^−4^*μ*M) treatment would inhibit P-P65 protein expression in LPS treated RAW264.7 cells [42]. The inconsistent responses may be due to the different doses of baicalin and species, which need our further research. Compared with the ETEC infection group, pretreatment with berberine hydrochloride significantly decreased the protein level of P-P65/P65 in the nuclear and decreased the mRNA expression of inflammatory cytokines (*IL-1α, IL-6, *and* CXCL-2*), indicating berberine hydrochloride can decrease the inflammatory responses of IPEC-J2 cells by inhabit the phosphorylation of P65 in the nucleus. Compared with the ETEC infection group, pretreatment with puerarin only decreased the mRNA expression of* CXCL-2*, showing puerarin cannot regulate the inflammatory responses of IPEC-J2 cells. The dosage variability of puerarin affects the absorption characteristics of baicalin [17], so puerarin can assist baicalin to play the anti-inflammation role in Gegen Qinlian Decoction.

In conclusion, pretreatment with puerarin, baicalin, and berberine hydrochloride reduced bacterial adherence by weakening cellular adhesion molecules and they improved the cellular morphology of IPEC-J2 cells. Pretreatment with berberine hydrochloride decreased the inflammatory response by regulating the NF-*κ*B signaling pathway in IPEC-J2 cells.

## Figures and Tables

**Table 1:** Primers used for real-time PCR.

Gene	*Primer sequence (5*′*-3*′)
	F: AGGATGCCCAATGGCTCTACT
*MUC4*	R: AAGGAGGCTGGTTCCGTTGAT
	F: GAGACTGGCTTTAGCAACCT
*MUC13*	R:AGTCTATCAAACCCTCACAC
	F: TAAGAATCTCAGAAACCCGAC
*IL-1α*	R: GGCTGATTTGAAGTAGTCCAT
	F: GAGAGCAATAAGGGAAATGTC
*IL-6*	R: TCTTCATCCACTCGTTCTGT
	F: TGCAGACCGTGCAAGGAATT
*CXCL-2*	R: TGGCTATGACTTCCGTTTGGT
	F: AAACCCTTCACTCCAGCACT
*PLAU*	R: TTGTCGGTACGGATCTTCAG
	F: GCTCTTCCAGCCCTCCTTCC
*β-actin*	R: ACAGCACCGTGTTGGCGTAG

## Data Availability

All the data in this study are available on request via corresponding author (Jianqin Xu, e-mail: xujianqincau@126.com).

## References

[1] Fairbrother J. M., Nadeau E., Gyles C. L. (2005). *Escherichia coli* in postweaning diarrhea in pigs: an update on bacterial types, pathogenesis, and prevention strategies. *Animal Health Research Reviews*.

[2] Zhu J., Yin X., Yu H., Zhao L., Sabour P., Gong J. (2011). Involvement of quorum sensing and heat-stable enterotoxin a in cell damage caused by a porcine enterotoxigenic Escherichia coli strain. *Infection and Immunity*.

[3] Devriendt B., Stuyven E., Verdonck F., Goddeeris B. M., Cox E. (2010). Enterotoxigenic Escherichia coli (K88) induce proinflammatory responses in porcine intestinal epithelial cells. *Developmental & Comparative Immunology*.

[4] Melkebeek V., Rasschaert K., Bellot P. (2012). Targeting aminopeptidase N, a newly identified receptor for F4ac fimbriae, enhances the intestinal mucosal immune response. *Mucosal Immunology*.

[5] Fleckenstein J. M., Hardwidge P. R., Munson G. P., Rasko D. A., Sommerfelt H., Steinsland H. (2010). Molecular mechanisms of enterotoxigenic Escherichia coli infection. *Microbes and Infection*.

[6] Nagy B., Fekete P. Z. (2005). Enterotoxigenic Escherichia coli in veterinary medicine. *International Journal of Medical Microbiology*.

[7] Ren W., Yin J., Xiao H. (2017). Intestinal microbiota-derived GABA Mediates Interleukin-17 expression during enterotoxigenic Escherichia coli infection. *Frontiers in Immunology*.

[8] Tang Y., Li F., Tan B. (2014). Enterotoxigenic Escherichia coli infection induces intestinal epithelial cell autophagy. *Veterinary Microbiology*.

[9] Ren W., Yin J., Duan J. (2014). Mouse intestinal innate immune responses altered by enterotoxigenic Escherichia coli (ETEC) infection. *Microbes and Infection*.

[10] Jacobsen M., Cirera S., Joller D. (2011). Characterisation of five candidate genes within the ETEC F4ab/ac candidate region in pigs. *BMC Research Notes*.

[11] Zhou C., Liu Z., Jiang J., Yu Y., Zhang Q. (2012). Differential gene expression profiling of porcine epithelial cells infected with three enterotoxigenic Escherichia coli strains. *BMC Genomics*.

[12] Zhou C., Liu Z., Liu Y. (2013). Gene silencing of porcine MUC13 and ITGB5: candidate genes towards Escherichia coli F4ac adhesion. *PLoS ONE*.

[13] Kim M., Ashida H., Ogawa M., Yoshikawa Y., Mimuro H., Sasakawa C. (2010). Bacterial interactions with the host epithelium. *Cell Host & Microbe*.

[14] Syed H. C., Dubreuil J. D. (2012). Escherichia coli STb toxin induces apoptosis in intestinal epithelial cell lines. *Microbial Pathogenesis*.

[15] Tang Y., Tan B., Xiong X. (2015). Methionine deficiency reduces autophagy and accelerates death in intestinal epithelial cells infected with enterotoxigenic Escherichia coli. *Amino Acids*.

[16] Li X.-Q., Zhu Y.-H., Zhang H.-F. (2012). Risks associated with high-dose lactobacillus rhamnosus in an *Escherichia coli* model of piglet diarrhoea: intestinal microbiota and immune imbalances. *PLoS ONE*.

[17] Kong H., Wang X., Wang Q. (2015). Effect of puerarin on the pharmacokinetics of baicalin in Gegen Qinlian Decoction () in mice. *Chinese Journal of Integrative Medicine*.

[18] Ling X., Xiang Y., Tang Q., Chen F., Tan X. (2017). Comparative pharmacokinetics of eight major bioactive components in normal and bacterial diarrhea mini-pigs after oral administration of Gegen Qinlian Decoction. *Journal of Chromatography B*.

[19] Zhang Y., Yuan J., Zhang Y. (2015). LC-MS/MS analysis of gegen qinlian decoction and its pharmacokinetics after oral administration to rats. *Biomedical Chromatography*.

[20] Zhou Y.-X., Zhang H., Peng C. (2014). Puerarin: a review of pharmacological effects. *Phytotherapy Research*.

[21] Chen J., Zhang R., Wang J. (2015). Protective effects of baicalin on LPS-induced injury in intestinal epithelial cells and intercellular tight junctions. *Canadian Journal of Physiology and Pharmacology*.

[22] Lim H. A., Lee E. K., Kim J. M. (2012). PPAR*γ* activation by baicalin suppresses NF-*κ*B-mediated inflammation in aged rat kidney. *Biogerontology*.

[23] Wang X., Zhao Y., Zhong X. (2014). Protective effects of Baicalin on decidua cells of LPS-induced mice abortion. *Journal of Immunology Research*.

[24] Liang S., Kuang Y., Ma F., Chen S., Long Y. (2016). A sensitive spectrofluorometric method for detection of berberine hydrochloride using Ag nanoclusters directed by natural fish sperm DNA. *Biosensors and Bioelectronics*.

[25] Cheng F., Wang Y., Li J. (2013). Berberine improves endothelial function by reducing endothelial microparticles-mediated oxidative stress in humans. *International Journal of Cardiology*.

[26] Zhang Y., Wang X., Sha S. (2012). Berberine increases the expression of NHE3 and AQP4 in sennosideA-induced diarrhoea model. *Fitoterapia*.

[27] Li R., Chen Y., Shi M. (2016). Gegen Qinlian decoction alleviates experimental colitis via suppressing TLR4/NF-*κ*B signaling and enhancing antioxidant effect. *Phytomedicine : International Journal of Phytotherapy and Phytopharmacology*.

[28] Wang Q., Song W., Qiao X. (2016). Simultaneous quantification of 50 bioactive compounds of the traditional Chinese medicine formula Gegen-Qinlian decoction using ultra-high performance liquid chromatography coupled with tandem mass spectrometry. *Journal of Chromatography A*.

[29] Ren J., Yan X., Ai H. (2012). Susceptibility towards Enterotoxigenic Escherichia coli F4ac diarrhea is governed by the MUC13 gene in pigs. *PLoS ONE*.

[30] Sheng Y. H., Lourie R., Lindén S. K. (2011). The MUC13 cell-surface mucin protects against intestinal inflammation by inhibiting epithelial cell apoptosis. *Gut*.

[31] Sun D., Courtney H. S., Beachey E. H. (1988). Berberine sulfate blocks adherence of Streptococcus pyogenes to epithelial cells, fibronectin, and hexadecane. *Antimicrobial Agents and Chemotherapy*.

[32] Luo H., Guo P., Zhou Q. (2012). Role of TLR4/NF-*κ*B in damage to intestinal mucosa barrier function and bacterial translocation in rats exposed to hypoxia. *PLoS ONE*.

[33] Luzio J. P., Pryor P. R., Bright N. A. (2007). Lysosomes: fusion and function. *Nature Reviews Molecular Cell Biology*.

[34] Hermes R. G., Manzanilla E. G., Martín-Orúe S. M., Pérez J. F., Klasing K. C. (2011). Influence of dietary ingredients on in vitro inflammatory response of intestinal porcine epithelial cells challenged by an enterotoxigenic Escherichia coli (K88). *Comparative Immunology, Microbiology & Infectious Diseases*.

[35] Wullaert A. (2010). Role of NF-*κ*B activation in intestinal immune homeostasis. *International Journal of Medical Microbiology*.

[36] Liu M., Song S., Li H. (2014). The protective effect of caffeic acid against inflammation injury of primary bovine mammary epithelial cells induced by lipopolysaccharide. *Journal of Dairy Science*.

[37] Xu X., Yin P., Wan C. (2014). Punicalagin inhibits inflammation in LPS-induced RAW264.7 macrophages via the suppression of TLR4-mediated MAPKs and NF-*κ*B activation. *Inflammation*.

[38] Da Silva J. B., Carvalho E., Covarrubias A. E. (2012). Induction of TNF-alfa and CXCL-2 mRNAs in different organs of mice infected with pathogenic Leptospira. *Microbial Pathogenesis*.

[39] Ismail H. A. H. A., Quan J.-H., Wei Z. (2012). Gene expression profiles in genetically different mice infected with Toxoplasma gondii: ALDH1A2, BEX2, EGR2, CCL3 AND PLAU. *The Korean Journal of Parasitology*.

[40] Wang X., Hardwidge P. R. (2012). Enterotoxigenic escherichia coli prevents host NF-*κ*B activation by targeting I*κ*B*α* polyubiquitination. *Infection and Immunity*.

[41] Yang X., Xiao Z., Liu F. (2016). Enterotoxigenic Escherichia coli infection alters intestinal immunity in mice. *Molecular Medicine Reports*.

[42] Cui L., Feng L., Zhang Z. H., Jia X. B. (2014). The anti-inflammation effect of baicalin on experimental colitis through inhibiting TLR4/NF-*κ*B pathway activation. *International Immunopharmacology*.

